# Using a socio-ecological framework to understand how 8–12-year-olds build and show digital resilience: A multi-perspective and multimethod qualitative study

**DOI:** 10.1007/s10639-022-11240-z

**Published:** 2022-09-30

**Authors:** Simon Patrick Hammond, Gianfranco Polizzi, Kimberley Jane Bartholomew

**Affiliations:** 1 School of Education and Lifelong Learning, University of East Anglia, Norwich, UK; 2 Department of Communication and Media, University of Liverpool, Liverpool, UK; 3 School of Education and Lifelong Learning, University of East Anglia, Norwich, UK

**Keywords:** Digital resilience, Socio-ecological, Children, Qualitative research

## Abstract

Educationalists’, researchers’, and policy makers’ work on children’s digital resilience has marginalised the role of the broader context within which digital resilience is constituted, experienced and derived. We aimed to address this lacuna by exploring how pre-teen’s digital resilience operates as a dynamic socio-ecological process. Addressing this aim, we employed participatory methods and thematically analysed eight focus groups with children aged 8–12 years (n = 59) and 20 telephone interviews with parents/carers and teachers of 8–12-year-olds and internet safety experts to examine this issue. We used purposive sampling and collected data over three months (January-March 2020). Our analysis constructed a matrix of main themes, constituent, and cross-cutting sub-themes. By placing this within a socio-ecological framework, we illustrate how pre-teens’ digital resilience operates within and across differing four levels (individual, home, community and societal) and four domains (learning, recognising, managing, and recovery). The paper advances the literature by illustrating how children can be supported to build and show digital resilience within and across different levels and domains. It is argued that digital resilience should be re-conceptualised as a collective endeavour involving children at an individual level, parents/carers within home environments, youth workers, civil society, teachers, and schools at a community level, along with governments, policymakers, and the education system and internet corporations at a societal level. We conclude by providing practice and research recommendations guiding those supporting children to facilitate opportunities to thrive online.

## Introduction

Learning how to recognise, manage, and recover from online risks is an increasingly important process for all. Digital resilience, which refers to this cyclical process, has been shown to moderate the negative impacts of risks whilst promoting opportunities to thrive online (Vissenberg & d’Haenens 2020; Vissenberg et al., 2022). Critically, exposure to risk is an antecedent of digital resilience. For educators then, the need to consider children’s risk exposure through a lifespan perspective warrants further examination (Hammond et al, 2022).

To better ensure children can benefit from the increasingly default digital interactions expected of all citizens, research has begun to try to unpick this dynamic process and illustrate the potential contributions of children’s, parents/carers’ and teachers’ derived risk and protective mechanisms in the face of online risks (Finkelhor et al., 2021; Haddon et al., 2020; Noll et al., 2021; Odgers & Jensen, 2020; Sage & Jackson, 2021; Sage et al., 2021; Stoilova et al., 2021; Tran et al., 2020; Valkenburg et al., 2022). However, our understandings of how children may (or may not) be supported to build, show and in some cases activate digital resilience and how this operates within and across different individual, home, community and societal contexts remains embryonic (Parry et al., 2021; Stoilova et al., 2021; Sun et al., 2022; Valkenburg et al., 2022; Vissenberg et al., 2022).

Knowledge is required to establish how children’s digital resilience is constituted, experienced, and derived from, complex relationships within and across differing interconnected and nested individual, home, community, and societal systems and how this evolves over time (Vissenberg et al., 2022). This is particularly important for ‘pre-teens’ (8-12-year-olds), who are transitioning into early adolescence and seeking more independence at home, school, within society and, increasingly, through online experiences. It is therefore vital that educators, who are progressively seen as playing a key role in supporting children to thrive in their connected lives (OECD, 2021), are equipped to assist pre-teens navigate these transitions.

We begin to address these gaps by exploring how digital resilience operates in relation to pre-teens’ use of the internet, and how parents/carers, educators, and civil society practitioners experience this through the introduction of a socio-ecological conceptual framework. We offer this socio-ecological conceptual framework of digital resilience to help parents/carers, educationalists, researchers, and policy makers locate and promote digital resilience opportunities across formal and informal education, acknowledging digital resilience as a process not a fixed outcome. Finally, we offer recommendations to facilitate ways to support pre-teens to pursue digital resilience opportunities, enabling them to thrive online.

## Theoretical background

### What is ‘digital’ resilience?

Broadly speaking, resilience is a process of recovering and/or growing from adversity (Southwick et al., 2014). It is studied by researchers from diverse disciplines with no consensus for an operational definition existing (Herrman et al., 2011). Once thought of as an enduring personality trait, the role of relationships within and across families, communities and societies in promoting resilience has widened its usage (Ungar, 2021). In this paper, we consider resilience as the capacity of an individual and/or system to respond and adapt positively to a stressor. Hence, we draw on understandings of resilience as a malleable process rather than an outcome, positing that digital resilience may operate similarly to psychological, family, community, and societal resilience.

To date, digital resilience is conceptually embryonic. The United Kingdom Council for Internet Safety (UKCIS) published their Digital Resilience Framework (DRF) (UKCIS, 2020), which typifies the current state of knowledge. The DRF consists of four process domains – i.e., “learn, understand, know, and recover” – but does not explain how these components were identified, nor if or how they operate on each other (UKCIS, 2020). So whilst the DRF is to some extent informative, like others in this area (Sun et al., 2022) it positions digital resilience as a ‘*personality’ asset* as opposed to considering how these process domains operate within and beyond individuals, thus lacking explanatory power and robustness of development (Vissenberg et al., 2022).

In summary, whether and how digital resilience transcends individuals to involve not just their home environments but also their communities and broader social contexts, and/or whether and how it operates within and across different process domains, warrants urgent attention if schools are to help children thrive digitally and pursue online opportunities (OECD, 2021).

### Translating knowledge of resilience across individual, home, community, and societal levels: A socio-ecological conceptualisation of digital resilience

Drawing on Ungar (2021), we place *digital* resilience within a four-level socio-ecological conceptual framework. This enables us to map and explore how each level operates within and across nested and interconnected systems at individual, home, community, and societal levels.

The first level considers individual resilience. Individual resilience research seeks to ascertain the intrapersonal susceptibility and protective factors assisting recovery and growth following adversity. Self-efficacy, optimism and emotional regulation are frequently cited as protective factors at this level (Ungar, 2021). In the context of digital resilience at this level, research efforts have focused on how ‘offline’ vulnerabilities including mental illness and special educational needs may contribute to greater risk exposure (El Asam & Katz, 2018; Livingstone et al., 2017), with work considering if better digital skills may help avoidance and/or responses to risk experiences (Haddon et al., 2020; Livingstone et al., 2021).

Research on resilience at the home level tends to focus on the concept of ‘family resilience’. Salient features of resilience at this level are access to supportive relationships capable of providing tangible and emotional support with warm, cohesive interactions (Khan & Deb, 2021). We recognise that such interactions are not just found in birth families, instead we use the label ‘home’ here to be more inclusive of diverse home environments. Digital resilience research at this level tends to emphasise parental mediation. Within this area, attention on parents’ mediation approaches and their impacts on children’s online access and experience (Chen & Shi, 2019; Samara et al., 2021) and digital skill development (Ren et al., 2022) have been considered. From a digital resilience perspective, an enabling (as opposed to restrictive) mediation style, encouraging resilience at a home level, increases children’s managed exposure to online risks, thus providing them with opportunities to be supported to build and show digital resilience (Livingstone et al., 2017).

Drawing on Pfefferbaum et al., (2017), resilience at a community level considers various interconnecting and interacting components, systems, structures, processes, and activities that encompass a given community. Resilience at this level comprises of social connectedness and social capital (Putnam, 2000). It is a process enacted to enable adaption that relies upon information and communication as well as upon the existence of a range of community capitals and agents to activate, learn from and resolve problems, take collective action, and transform (Pfefferbaum et al., 2017).

Digital resilience at a community level broadens understandings of communities as defined physical spaces and becomes related to the digital literacy skills, knowledge, experiences, and mediation approaches of those within an individual’s support network(s), as well as the links between and within these networks. Work in this area highlights the differing roles of social capital for vulnerable users (Hammond & Cooper, 2015; Hammond et al, 2022; Marler 2021), the increasing role played by educators as mediating community members (McDonald-Brown et al., 2017) and how pressing social issues such as digital exclusion function as a result of other economic, cultural, social and personal inequalities that can impact on opportunities for children to be supported to build and show digital resilience by members of their networks (Helsper & Eynon 2013).

Societal resilience is the final level and is a process through which systems operate before, during and after threats. This process relies on the capacity of societies to prepare, prevent, and protect before disruption, to mitigate, absorb and adapt during disruptions, and to restore, recover and transform after disruptions (Linkov & Trump, 2019). Digital resilience at this level relates to key societal actors such as governments, internet corporations, the education system, civil society, and the cultural norms these actors draw upon before, during and after perceived threats.

### The present study

Although research has focused on each of these levels, there is a dearth of literature on the ways in which these levels intersect. With a primary emphasis on digital resilience as a socio-ecological concept, this article begins to address this lacuna. This is important for educators as schools have in increasingly central role in shaping how children play, learn, and grow online (OECD, 2021). Hence, whether and how digital resilience transcends individuals, their home environments, their communities, and broader social contexts, and/or whether and how it operates across different process domains, warrants urgent attention if schools are to enable children to digitally thrive. Our research questions therefore ask:

1. *How does digital resilience operate in relation to how pre-teens use the internet?*
2. *How do parents/carers, educators and civil society practitioners experience pre-teens’ building and showing of digital resilience?*

## Methods

### Design and participants

The project was aligned with social constructivism, approaching digital resilience as contextually situated (Gergen, 2015). It employed a mixed qualitative methodology with data collected from pre-teens via focus groups and from adult stakeholders via telephone interviews over three months (January–March 2020).

Fifty-nine pre-teens, (23 males and 36 females, *M* age = 11.16 years, range 8–12 years) from six schools from across three English regions (two per area from East Anglia, East Midlands, and Greater London) participated, with a total of ten focus groups held within these schools. Of the ten focus groups, nine were conducted with the support of, or led by, a Young Person Co-Researcher (YPCR). In the context of this paper, six YPCRs (aged 16–17, three females and three males) were recruited from high schools located within the same regions as the focus groups. We trained YPCRs in relevant ethics, focus group facilitation and data analysis techniques. During data analysis, as outlined below, YPCRs contributed to the ongoing refinement of the coding framework established, bringing their ‘insider knowledge’ (in comparison with that of the researchers) to this process.

To explore the views of relevant adults, we also undertook telephone interviews with 20 adult stakeholders (ten parents/guardians and six education and four civil society practitioners with expertise in Internet Safety Education (ISE)) from across the UK. Of these adult participants, ten were female and ten were male (for more information on the overall sample, see Table 1).

Study methods and results are reported according to the Consolidated Criteria for Reporting Qualitative Research (COREQ) (Tong et al., 2007). See Appendix 1 for COREQ checklist.

### Ethical considerations

In asking pre-teens and adult stakeholders to share their experiences of online risks, ethical considerations were imperative, especially considering the involvement of YPCRs in the focus groups. We mitigated risks by training and supporting YPCRs in relation to ethics and offered appropriate pre and post focus group supervision. To avoid potential confidentiality issues (for example YPCRs knowing pre-teen participants and/or their families), YPCRs’ schools were sufficiently distant. Pre-teens themselves were made aware that safeguarding concerns would be passed on and handled according to in-situ school procedures, with this reiterated prior to conducting the focus groups. For all participants, age-appropriate resources to assist online risk management were offered if the researcher felt this was required. All data was pseudonymized at the point of transcription.

Ethical approval was provided by the School of Education and Lifelong Learning Research Ethics Committee at the University of East Anglia [Ref: 2019/09/SH].

**Table 1:** Participant demographics

	Pre-teen (n = 59)*	Adult Stakeholders (n = 20)
Mean age (years)	11.16	40.21
Gender		
Male	23 (38%)	10 (50%)
Female	36 (62%)	10 (50%)
Ethnicity		
Asian or Asian British - Bangladeshi	2 (3.4%)	0
Black or Black British - African	3 (5.0%)	0
Mixed - White and Asian	0	0
Mixed - White and Black - Caribbean	2 (3.4%)	0
Mixed - White and Black – African	0	0
Other	4 (6.7%)	1(5%)
Other Black background	2 (3.4%)	
Other White background	9 (15.25%)	1 (5%)
Prefer not to say	3 (5.0%)	1 (5%)
White British	34 (57.62%)	16 (80%)
White – Irish	0	1(5%)
Highest education level reached		
Further Education (e.g., A levels)	NA	1 (5%)
Undergraduate degree	NA	9 (45%)
Postgraduate degree	NA	10 (50%)
Teacher participant school setting		
Primary	NA	1 (20%)
Secondary	NA	2 (40%)
Teachers across both primary and secondary education settings	NA	3 (60%)

### Sampling and recruitment

Purposive sampling was used. Participants were recruited across dimensions of diversity including different organisational/professional contexts and different household contexts. Sample heterogeneity was sought in terms of age, gender, socio-economic status, and ethnicity. Schools were approached through the authors’ networks with a view to ensuring diversity of geographical locations across the urban-rural continuum. Pre-teen participants were recruited through their schools, with recruitment packs (containing parent/carer and pre-teen participant information sheets, parent/carer consent forms and pre-teen approval forms) sent home. Six-hundred and twelve pre-teen recruitment packs were sent out, with 63 returned (four pre-teens were unable to participate as parent/carer consent forms were not returned).

Adult stakeholder participants were recruited via various strategies to assist diversity, including via existing networks, snowballing, social media, newsletters, and blog posts.

### Data collection

Pre-teen focus groups took place in a quiet/private location in schools. These involved one researcher – either SH or GP – and YPCRs (maximum of two YPCRs per focus group). Upon attending each school, the researcher held a briefing with YPCRs regarding how the focus group would unfold and who would play each role (administer/note taker, chair, supplementary questioner). The researcher would check appropriate ethical forms before pre-teen participants entered the room. Pre-teen participants were then briefed on the boundaries of the focus groups before they began. Pre-teens were asked to share and discuss occasions they had been online, come across risks and/or made mistakes and how they and those around them responded. At the end of the focus groups, pre-teens were invited to ask questions before being debriefed and thanked and returned to class. Focus groups were 45–60 min long. Additional details of focus groups are provided in Table 2 below.

**Table 2:** Details of focus group locations

Focus Group (FG) number	School number	Region
FG1	1	Yorkshire/East Midlands
FG2	2	Yorkshire/East Midlands
FG3	2	Yorkshire/East Midlands
FG4	3	London
FG5	4	London
FG6	4	London
FG7	5	East Anglia
FG8	5	East Anglia
FG9	6	East Anglia
FG10	6	East Anglia

Adult stakeholder telephone interviews were undertaken by researchers. At a mutually convenient time, the researcher called the participant, requested confirmation that they had understood study information, addressing any questions raised. The researcher then turned on the dictaphone, took verbal consent and undertook the interview. Adults were asked to discuss occasions they had experienced pre-teens being online, come across risks and/or made mistakes and how they and those around them responded.

At the close of the interviews, participants were invited to ask any questions, debriefed, and thanked. Interviews were 30–45 min long.

Data collection team were SH, an applied psychologist qualified to PhD level, and GP, digital literacies researcher, qualified to post-graduate level at the time of data collection. No pre-teen participants were known to the team at the time, but some adult stakeholders were. Participants were fully aware of the goals of the research. YPCRs assisted in the development of the focus groups questions and helped with piloting by providing feedback on the topic guide designed for the focus groups. Due to the data collection methods used, adult stakeholders were offered the chance to review their transcripts, pre-teens were not. One adult participant did this, but no changes were requested.

### Analytical procedure

Drawing on Braun & Clarke’s (2006) six phases of thematic analysis, focus group and interview data were transcribed verbatim, anonymised at the point of transcription and coded in Nvivo. A thematic approach was selected as we sought to provide a rich and detailed, yet complex, account of patterns of meaning across the data corpus. Following initial readings of a random 20% sub-set of transcripts from differing participant groups by SH and GP and YPCRs, an inductive coding framework was co-developed with YPCRs over a series of three meetings. At these three roundtable meetings, responses from pre-teens and adults were reviewed and discussed to enable rich multi-perspective conceptualisations as opposed to what pre-teens or adults in various roles thought in isolation.

Coding was then undertaken by GP with regular conversations and meetings held with SH to further clarify nuances. During these analytical processes, SH and GP developed themes that were sensitive to, as opposed to being guided by, our shared and evolving understanding of digital resilience as a socio-ecological concept. In other words, the potential usefulness of a socio-ecological framework to map talk and experiences was constructed organically during the analytical process as opposed to being developed before and applied deductively from its commencement. This framework was then used by SH and GP to code the remaining data with subsequent discussions held with YPCRs to enhance analysis via their ‘insider’ perspective.

Utilising this increasingly abductive approach enabled researchers SH and GP to apply initial inductive codes to test and refine the developing socio-ecological conceptual understanding and then move back and forth between data and theory iteratively. Coded data were then re-examined, and key themes extracted for each level of resilience (i.e., individual, home, community and societal).

Our coding of process domains was more deductive initially as we sought to test out the suggested component processes posited by the UKCIS DRF (UKCIS, 2020). As outlined previously, the DRF uses the phrase ‘learn, understand, know, and recover’ to articulate components of digital resilience as a dynamic process (UKCIS, 2020). The labels applied to the process domains developed by our analysis (i.e., learning, recognising, managing, and recovery) were guided, but not limited, by the DRF to advance, rather than being restricted by, previous knowledge. These are also the terms which resonated with the empirical data we collected and our conversations with YPCRs.

## Findings

Across the data set, our analysis demonstrated how digital resilience operated at different levels (i.e., individual, home, community and societal) and involved different domains (learning, recognising, managing, and recovery) which operated within and across these different levels.

Due to the focus of the paper, we orientate each level of digital resilience (i.e., individual, home, community and societal) as a main theme with each having four cross-cutting sub-themes (i.e., learning, recognising, managing and recovery) with illustrative summative descriptions provided in Table 3. It is important to note that the ways in which pre-teens’ digital resilience operated across these different levels and domains are not mutually exclusive but reinforce and operate on and with each other. We will now present findings for each level of resilience and, within these, each process domain.

**Table 3:** Main themes and constituent and cross-cutting sub-themes showing how participants conceptualised digital resilience within a socio-ecological framework

	Individual Level	Home Level	Community Level	Societal Level
Learning Domain	Learning via maturation and experience	Learning scaffolded by home environments	Learning via and within cohesive communities and networks	Learning via activating societal systems and shared responsibility and accountability
Recognising Domain	Adults more anxious than pre-teen, but pre-teens can recognise when they feel anxious	Open conversations within home environments can increase risk recognition	Cohesive supportive and support networks assist risk recognition	Recognition is increased as societal systems adapt to risks and provide risk recognition opportunities
Managing Domain	Experience helps pre-teens when navigating risks practically and emotionally	Open and supportive conversations in home environments aid management	Cohesive supportive and support networks assist risk management	Managing is reinforced when systems promote safety information and safety by design
Recovery Domain	Recovery is retroactive and involves acceptance but not always growth	Recovery opportunities mediated within and by home environments	Recovery opportunities mediated via and within community networks	Recovery opportunities mediated by systems with shared responsibility and accountability

### Pre-teens’ digital resilience at an individual level

At this level, digital resilience emerged as related to pre-teens’ capability to self-protect. Participants’ views of what facilitates digital resilience at this level included pre-teens’ developmental maturity, emotional regulation, agency and, in agreement with Sun et al., (2022) pre-teens’ self-efficacy.

#### Learning at an individual level

Extract 1:

‘I don’t think they are ready to be their best selves online…we need to wait…until they develop more empathy…’ (P01_Parent).

Extract 1 shows how adults viewed pre-teens’ capability to learn how to use the internet in ways that relate to their ‘emotional maturity’ (‘empathy’) as part of an age-related developmental process rather than experiential learning. In this way adults primarily constructed pre-teens’ learning about online risks at the individual level via conventional understandings of childhood maturation Yet, pre-teens discussed how they learned how to navigate different online contexts experientially:

Extract 2:

Researcher: If you were to report something…would you know how?

Alice: Maybe.

Ben: I would know.

[Agreement]

Ben: I would know, because if it’s a new game then you probably won’t know how to do it but if you’ve played it for ages, you’d know. (FG2).

Extract 2 shows how pre-teens’ capability to develop digital resilience depends on their exposure to familiar contexts underpinned by experiential learning. As such, we can begin to see how adults primarily constructed pre-teens’ learning about online risks at the individual level via conventional understandings of childhood maturation. However, as with previous research (Dutton & Shepherd, 2006; Hurwitz & Schmitt, 2020), our analysis illustrates that learning about online risks is also an experiential process. A tendency for taking a protectionist approach to online risks derails opportunities to develop digital resilience via risk exposure. In agreement with previous research, by providing less learning opportunities as opposed to more, adults prioritise short-term risk reduction over digital resilience as a lifelong pursuit (Hammond and Cooper, 2015).

#### Recognising at an individual level

Recognising risks is vital for moderating online risk experiences and at an individual level is oriented towards understanding online risks in ways that are grounded in emotional responses. Adults often reported being concerned about pre-teens not having the capability to spot hidden online risks that they could. The inference here is that adults can recognise risks in ways that pre-teens cannot:

Extract 3:

‘My concern is him being exposed to videos that he would find distressing…I think that’s because he doesn’t really know what’s out there, he worries less about it than I do…’ (P19_Parent).

Whilst recognising that pre-teens may be exposed to online risks is emotionally burdensome for adults, pre-teens’ experiences of coming across online risks, regardless of whether they recognise these or not, had emotional impacts:

Extract 4:

Researcher: How do you feel about…Apple, or whatever company…having bits of your data?

Carl: It’s a bit weird really, because there’s this game, I deleted it, then like a year later…I was on the same level and it was freaking me out…it remembered the name, my number, the shirt…everything… (FG10).

Here our analysis highlighted that, although adults are often portrayed (by themselves and/or others) as lacking digital literacy skills and knowledge, they legitimise their emotional distress on assumptions that they recognise more online risks than pre-teens (Livingstone et al., 2017). Regardless of recognition, pre-teens often reported their experiences of online risks as distressful, illustrating the need to focus on navigation and recovery (Wright, 2016) and in turn echoing the need to shift towards examining how children engage with their worlds in ways that are mediated by the internet, as opposed to how they engage with the internet (Livingstone et al., 2018).

#### Managing at an individual level

In agreement with previous research, managing risks at an individual level was characterised by pre-teens sharing what they do agentically, practically and emotionally to manage online risks themselves (Buchanan et al., 2017; Wright, 2016). Data in this cross-cutting sub-theme demonstrated digital resilience at an individual level as enacted in response to perceived disruptions or threats. Strategies used by pre-teens included ignoring or disengaging, with Extract 5 offering a rationale for this:

Extract 5:

Researcher: You said you felt a little bit scared because you saw…an ad about people murdering each other…what did you do?

Sangeet: I went onto Kids YouTube instead….

Researcher: And you didn’t talk to…your parents?

Sangeet: No…because I didn’t think it was something I needed to talk to them about. (FG5).

#### Recovery at an individual level

Recovery at this level was described by pre-teens in ways that suggest acceptance of, and growth from, experiencing and either recognising and/or managing risks independently.

Extract 6:

Kai: I reported somebody before, and they were banned from the app.

Researcher: And does that make you feel…‘safer’?

Bella: It makes me feel safer.

Kai: Yeah, because I know that they won’t do anything again because they won’t be able to play it. (FG9).

As a result of proactively acting in the moment, pre-teens managed to retroactively learn about navigating risks to grow from experience and avoid these in the future, thus developing feelings of recovery. This suggests that the process of developing digital resilience at this level relies on an understanding of childhood as powerful and agentic (Valentine, 2011).

Extract 7:

Johan: I was a pretty scared when it was happening but then afterwards, I was like proud of myself for sorting it out and like reporting them.

Researcher: What about you?

Emily: Yeah, sort of, I still told my mum about it afterwards, but it was nice to be able to like you know like to handle it myself like at the time (FG6).

Drawing on mental health understandings of trauma and post-trauma growth (Seligman, 2012; Tedeschi & Calhoun, 2004), our analysis indicated two recovery processes following online risk exposure, acceptance and growth. Acceptance alone indicated a returning to the previous *status quo*, (shown in extract 6) whereas, drawing on ideas of post-traumatic growth (Tedeschi & Calhoun, 2004), growth indicated positive change because of a risk experience, as evidenced by Extract 7. This also illustrates the interactions between the differing levels of digital resilience and how they can operate on how incidents are experienced and reflected upon.

### Pre-teens’ digital resilience operating at a home level

At this level, our analysis illustrates how pre-teens build and show digital resilience in ways that depend on their relationships with those within their home settings (typically, but not exclusively, parents/carers and siblings), and across domains of learning, recognising, managing, and recovery.

#### Learning at a home level

Pre-teens’ ability to learn how to build and show digital resilience was intertwined with mediation practices and scaffolded by relationships in home environments:

Extract 8:

‘My son…has got a real thirst for learning that enables him…when we look at [information online] together, to really enhance his knowledge and see different perspectives…so I can see a benefit…under our supervision because…if you Google certain words, anything can come up’. (P10_Parent).

Here we can see how curiosity enables this pre-teen to use the internet constructively to search for information for educational purposes, but only when coupled with parental monitoring. In this way, risks are reframed as learning opportunities.

Based on the cultivation of supportive relationships and open conversations, this process of scaffolding is characterised by enhanced trust. It encourages pre-teens to share their online experiences with those trusted within home settings. Simultaneously, it contributes to their development as responsible users, concurrently encouraging trusted others to adopt more enabling practices of mediation:

Extract 9:

‘Online…[my son]…often tells us of friends that have used…words that he shouldn’t…so we do feel like he is quite. mature…in that respect. That is why I probably give him slightly more allowances because I do trust him, because…he’s more mature than most of his friends’. (P13_Parent).

In line with previous research, this role, usually fulfilled by parents/carers, can facilitate or impede experiential learning opportunities (Livingstone & Helsper, 2008; Livingstone et al., 2017). In the context of this paper, this is viewed as impacting on how pre-teens may build and show digital resilience, thus illustrating the need to focus learning resources on home environments, not just individual pre-teens in isolation.

#### Recognising at a home level

Pre-teens’ capacity to build and show digital resilience relies on the extent to which they can recognise different online risks. Tangible support from trusted others within the home was experienced as enhancing recognition at this level:

Extract 10:

Fraser: I have an older brother my parents to be fair kinda tell me about the mistakes he’s made and like what to look for so I can like recognise things.

Ah Lam: Like that aren’t true?

Fraser: Yeah, but like also how not to do some of the stuff he done. (FG2).

Having conversations with pre-teens that enable them to gain awareness of risks was seen as important for pre-teens and parents/carers in enabling pre-teens to build awareness of online risks and implement strategies to manage risk. These intertwined processes, again, depend on pre-teens’ opportunities to engage in open dialogues in home environments that recognise digital resilience as a lifelong process:

Extract 11:

“I think they need to know accidents happen, and curiosity happens but that’s okay, but they need to be able to report it…”. (P19_Parent).

As outlined in extract 11, home environments may operate optimally when they allow emotionally supportive and non-judgemental dialogues.

#### Managing at a home level

When managing at the home level, pre-teens provided accounts of when parents/carers assisted practically and emotionally:

Extract 12:

Amber: I know what the password is, I first I have to ask my mum of, ‘Can I watch this series?’ and then she checks if it’s scary. Like, my mum’s watching Stranger Things and she’s waiting ‘til like I’m a bit old enough and then when she’s finished the whole series, she said, ‘Right, okay, you can re-start it and I will sit with you if you like.

Charlie: My parents don’t really allow me to go on YouTube and, on my iPad they have like this ‘kids’ account and they have kids YouTube and if I see anything that’s I don’t think is right, they say that I should tell them and then they’ll do something about it. (FG5).

Extract 12, in agreement with the parental mediation literature (Haddon & Livingstone, 2017), shows how pre-teens’ management of online risks at a home level was underpinned by their ability to have open non-judgemental conversations with trusted others in combination with relationship based mediation practices. Building on this work across our data corpus, pre-teens also noted there were thresholds for seriousness that were balanced against pre-teens’ awareness of the need to sacrifice privacy to activate differing assets in home environments.

Extract 13:

Lucy: If I see something I don’t like feel comfortable seeing, cos I don’t really trust my parents they’ll just take my iPad…so, I try to tell my sisters.

Milly: I just like turn it off and try not to watch it again or like watch Cat videos to cheer me up.

Max: I can’t really do that cos I ain’t got a sister or nothing.

YPCR: So what do you do?

Max: Erm, just sort of like, well if it’s bad I kinda go to my mum if it’s not to like awkward but if it is I switch it off or like talk to my mate or his mum if it’s really bad. (FG1).

In line with previous research, some pre-teens described how they would turn to siblings because of their own privacy concerns and believed that parents/carers would remove access instead of providing support (Smahel & Wright, 2014). In our analysis, Extract 13 illustrates how pre-teens made contextually situated decisions in relation to how to manage risk exposure and which level of digital resilience to activate (in this case individual, home or community). We extend this knowledge by illustrating how a socio-ecological perspective enables digital resilience capacity (number and types of relationships) as well as capability (to implement both skills and/or knowledge) within pre-teens’ networks to be considered more closely, something discussed further below at the community level.

#### Recovery at a home level

Recovery at this level was constructed as reliant upon their relationships and the support these provided. A key aspect of recovery at the home level appears to be pre-teen’s acceptance of, and growth from, negative online experiences, mediated by tangible and emotional support from home environments.

Extract 14:

Jon: Well for me when it all kicked off, I was freaking out and after like it had calmed down….

Researcher: After it had calmed down?

Jon: I guess like how they [parents] helped me sort of handle and talk to the school it made it easier to like deal with it and like it’s like sick.

Researcher: Sick?

Jackson: [laughing] Like sick! You know like good!

Jon: Yeah, like it was sick to know they were sort of there and they got it, you know and that listened, and it wasn’t something I thought they’d deal with but they did and that was good. (FG7).

Acceptance at this level was constructed by pre-teens as an understanding that something too risky had happened that had emotional repercussions but that things had returned to normal with the support of others in their home environment. As Extract 14 illustrates, growth at this level was framed by pre-teens’ reflections on, and increased awareness of, the capability to call upon positive coping mechanisms (i.e., empathic parental support). Here growth appears to be a product of learning and recognising how to manage online risks through having recovered from these, thus suggesting digital resilience development may be a cyclical process across process domains that is mediated within and across differing levels.

### Pre-teens’ digital resilience at a community level

Pre-teens’ capability to activate digital resilience at a community level relied on the capacity to use, and the extent to which they had access to, cohesive resources that could enable them to learn, recognise, manage, and recover from online risks in ways that were mediated by key community actors such as wider family members, peers, and teachers and institutions such as schools (henceforth, key community actors).

Drawing on Pfefferbaum et al., (2017), this means that digital resilience can only circulate at this level as a collective property that transcends the individual as long as it rests on social capital – that is, the social relationships and connections that characterise a particular community (Putnam, 2000).

#### Learning at a community level

Pre-teens’ capability to learn how to develop digital resilience is grounded in formal ‘top-down’ and informal ‘bottom-up’ networks. These networks enable key community actors to develop the skills and knowledge needed to provide pre-teens with supported opportunities to learn:

Extract 15:

‘…you often feel you’re one step behind…so I ask [school] colleagues or colleagues with children who know or sometimes you just ask the children.’ (P17_Teacher).

Extract 15 illustrates how communities may learn to build digital resilience capabilities through formal and/or informal learning. Hence the capacity of communities to assist pre-teens to learn about online risks is rooted in the networks within, and between, key community actors serving as resources of tangible support and knowledge.

#### Recognising at a community level

At this level, adults who represented key community actors in pre-teens’ communities shared ways in which they sought to improve recognition skills and knowledge via two routes. Firstly, indirectly via increasing a community’s recognition capacity in relation to risks by increasing knowledge within networked actors:

Extract 16:

‘…my focus…when I am working with adults, is for them to recognise that…it’s…more…about who are they [pre-teens] following, who is influencing them, what sort of space are they getting themselves into’ (P11_Civil Society Practitioner).

Secondly, more directly via encouraging pre-teens to access their community’s capabilities and use these resources to assist them to develop digital resilience:

Extract 17:

‘…when incidents happen, they [pre-teens] need somebody trusted to speak to…adults can have really different views about it…and sometimes that tension just means that they just won’t ask…it is not always a teacher [or] parent that can play that role…but often other family members or other people in the community who have a more informal relationship…’ (P15_Teacher).

Here our analysis extends existing knowledge in this area by illustrating how access to informal and formal resources shared by key community actors can increase pre-teens’ and trusted others’ recognition of online risks, thus underlining the need to improve the evidence and training available to key community actors (El-Asam et al., 2021) including educators who have an increasingly central role in shaping how children play, learn, and grow online (OECD, 2021).

#### Managing at a community level

Pre-teens’ capability to manage online risks is also shaped by access to support and resources within their communities. This can include access to formal education provided via schools:

Extract 18:

Amelia: We did have Safer Internet Day.

YPCR: So, what did you do?

Amelia: We made our own characters and then we talked about how they make us safe and feel safe.

Anna: And how our identity is safe online with other people.

YPCR: So how do you keep your identity safe online?

Leroy: Well, what I do is, I make my character not look like me. (FG9).

Extract 18 indicates how an annual online safety campaign ‘Safer Internet Day’ delivered information to pre-teens via community members, in this case via schools as organisations accessing and implementing resources. Conversely, Extract 19 illustrates how members of pre-teens’ networks can also access community resources to aid pre-teens’ ability to build and show digital resilience by seeking advice on management strategies developed through experience:

Extract 19:

‘I’m probably going to have a conversation with the school mums and dads…and see if there’s anything they have…because I always find they have the best advice…to see what they’ve got in place and what they do…’ (P18_Parent).

Here we see that, in line with previous research, pre-teens reported drawing on formal and informal education and support from within their communities to manage their negative experiences (Buchanan et al., 2017). In advancing this work, our analysis illustrated the extent to which pre-teens indirectly, via their trusted others accessing support from key community actor networks and assets, also helped to build and show digital resilience. This indicates that resources need to target not just children or home environments but the wider ecosystems within which pre-teens interact and are enmeshed. In short, individual, home, or societal level sources of digital resilience may remain suboptimal when key community actors and networks remain inactive.

#### Recovery at a community level

Recovery was experienced when communities adjusted to return to a required level of safety. Importantly, communities could be successful in one aspect of recovery (e.g., acceptance of events) but without necessarily succeeding in another (e.g., growth):

Extract 20:

‘…we get a lot of parents…sending us screenshots of things that have been said between pupils… on WhatsApp…we obviously just reply “Well, that’s below the restricted age they should not be on it and, yes, we will talk to them…” I think it’s an un-treaded path for schools as to whether they have the right to say…“You shouldn’t be on WhatsApp, you’re too young.’ (P03_Teacher).

Extract 20 illustrates how key community actors may accept events but not grow from experiences. Here, risk recovery is constructed as a single episode rather than longitudinally (i.e., post-risk growth), particularly when chronological age is no longer a valid restriction rationale. Extract 21 indicates how pre-teens experience such approaches and how this may reduce pre-teens’ capacity to call on key community actors, such as schools, to help digital resilience development:

Extract 21:

Zak: they [teachers] shouldn’t have really done that assembly…arguing with us because I feel like they should have just done a letter saying like, “Just to make you aware that your children have TikTok…” just to make parents aware…they really shouldn’t have shouted at us because it’s not really fair…it’s our parents’ decision….

Chloe: And it’s not their choice what apps we have….

Lizzie: They can’t like tell us that we can’t have it if it’s not up to them… (FG1).

Pre-teens’ recovery opportunities were impacted by their community capital, synergies and tensions between the mediation strategies of key community actors and across levels. Our analysis illustrates how tensions between levels (e.g., home and community) can set up conflicts that place pre-teens in positions that compromise their longer-term capacity to build and show digital resilience. Consequently, each pre-teen’s individual community of support is likely to operate differently depending upon their contexts and experiences. Discontinuity across contexts appears likely to provide suboptimal support, again, underlining the contribution of a socio-ecological understanding of digital resilience.

### Pre-teens’ digital resilience at a societal level

Digital resilience at this level emerged as pre-teens’ capacity to seek support from civil society, governments, dominant cultural norms, and beliefs. A clear message across process domains was the belief that ‘intervention’ on a more systemic level would impact individual pre-teens’ digital resilience, their home relationships, communities, and societies as sources of digital resilience, as well as the interactions amongst these levels.

The timeframe at which data collection occurred (January – March 2020) enabled participants’ experiences of societal reactions to the Momo hoax (circa March 2019) and the then advancing COVID-19 pandemic (March 2020) to act as ‘theoretical consoles’, that is, phenomena that compel us to propose, interrogate and theorize (Verhoeff, 2009). This meant the ways in which digital resilience operated at societal level and across domains could be explored via participants’ experiences of these phenomena retrospectively (the Momo hoax) and prospectively (COVID-19).

#### Learning at a societal level

Learning at this level was discussed in relation to the roles, responsibilities and different assets stakeholders may rely upon or activate in relation to supporting pre-teens to learn about risks in an equitable manner. At this level, pre-teens were positioned as needing support from governments, internet corporations, education systems and civil society (henceforth ‘key societal actors’):

Extract 22:

‘There is the child, but…people must take responsibility for their children. You can’t put all the onus on the government…or…a company…Things are still going to happen, and people need to be educated…I think that’s where the government should come into play…Parents… [should] be offered [classes] in the community…’ (P04_Teacher).

Here Extract 22 illustrates the complexity that characterises how learning at this level was experienced, and the interconnectedness of shared responsibilities for providing learning opportunities at this macro-level. This is examined further by Extract 23:

Extract 23:

Amelia: Our school, like with all that Momo stuff, started sending more letters home our parents…they never used to do that much before….

Leroy: (laughing) My cousin’s school were the same, like all the schools were wigging-out it weren’t even real!

Bee: And they’ve [teachers] been saying to like to come to them if we’re worried.

Amelia: Worried?

Bee: By all this China and Italy virus stuff so they can sort of talk about it all with us and use it in our Fake News assembly. (FG9).

Here learning at a societal level is discussed in the context of pre-teens’ experiences of the education systems’ response to the disruption, mitigation and transformation following the Momo hoax of Spring 2019. Pre-teens reflected on how their experiences of the education system were enacted via increased communication between community and home levels ‘more letters home’ and via the education system activating key community actors (in this case educators) to promote formal learning opportunities via ‘Fake News assembly’.

#### Recognising at a societal level

At this level, dominant cultural norms of working towards the common good, and the need for equity in assisting pre-teens to build and show digital resilience resonated. Pre-teens described how schools and parents offered differing views with adults, describing how key societal actors had interconnected responsibilities to find better ways to promote digital resilience by providing parents/carers with the support they needed to better recognise online risks and facilitate, in turn, pre-teens’ digital resilience:

Extract 24:

’It is a system’s thing really isn’t it. The classic is like whenever a new crazy like MoMo appears, the people who run platforms have a responsibility…to make things safer and take stuff down, Government may need to respond or should have ways to respond, community settings like schools, the kids and their parents’ all have a responsibility…but there has to be more equity, many parents, families and well teachers can be quite computer illiterate, I think that it’s important that people realise how little adults really know… their messages need to be really simple and understood by all’. (P16_Civil Society Practitioner).

This is important in demonstrating the value of identifying strengths and weaknesses in pre-teens’ differing levels of digital resilience to optimise child-centred support. As research illustrates, digital exclusion is often the result of economic, cultural, social and personal inequalities (Helsper & Eynon, 2013). Hence, we need to recognise that equitable digital resilience support cannot rely on a ‘one-size-fits-all’ approach.

#### Managing at a societal level

At this level, the ways in which pre-teens’ negative online experiences can be managed were illustrated in participants’ descriptions of occasions when they had, or intended to, access support from key societal actors on how to manage online risks. Critically, this was orientated by participants to enable enjoyment opportunity as opposed to simply orientated around safety:

Extract 25:

‘Who has a responsibility? Umm, our governments and…all within the tech ecosystem have a responsibility and the responsibility that we all have in society…making the internet a place where they [pre-teens] can thrive and take advantage of it all’. (P05_Civic Society Practitioner).

Pre-teens discussed the responsibilities of key societal actors including, in this case, internet corporations in terms of managing to make their online experiences enjoyable:

Extract 26:

[YPCR] If there was something you could change about the internet, what would it be?

Maurice: Adverts!

Emily: Yes!

Johan: Adverts.

Alexia: Ads.

Davina: Ads that are inappropriate and stuff.

Emily: No ads, please, delete them!

Harry: Yes, they annoy you when you’re trying to play!

Emily: Yeah, or they’re really inappropriate…and they waste my time!

Teddy: I’ve had games where they’re like 3 + and then there’s been like a 15 + game advert come on…it’s not good. (FG6).

Here we see how societal level digital resilience is orientated as more than solely managing risks, but about enjoyment and thriving. This is an important consideration, and one in which tailored support to optimise pre-teens resonates.

#### Recovery at a societal level

Recovery at this level was framed by participants through wider cultural norms and beliefs regarding the roles and responsibilities of key societal actors to help pre-teens recover to thrive online after risk exposure.

Extract 27:

‘…it’s about what happens next, so they’ve made a mistake or whatever, you know what do we do next, as a society, the platforms, government and education system, schools and parents and also the kid, how to we help them [pre-teens] learn from mistakes? It’s not just banning it, who does that help? We don’t ban them from football if they fall down do we?’ (P03_Teacher)

As illustrated in Extract 27, pre-teens’ capacity to recover from online experiences depends on key societal actors and the interplay of these actors in promoting digital resilience opportunities across levels. Enabling recovery following negative online experiences is mediated by broader socio-cultural processes. The extract also highlights the overlapping nature of recovery and learning process domains within and across the differing levels of digital resilience. Again, our socio-ecological framework illustrates the complexity and interplay of the constituent components of digital resilience, thus reinforcing its usefulness in advancing the conceptualization of digital resilience.

## Discussion

This important paper represents a vital step in developing the knowledge base underpinning digital resilience as a socio-ecological concept. By locating its analysis within a socio-ecological framework (Ungar, 2021), the paper illustrates how displaying and activating digital resilience is a power-ridden process, mutually constituted by top-down and bottom-up forces. The paper illustrates that pre-teen digital resilience operates across different levels (individual, home, community and societal) and domains of learning about, recognising, managing, and recovery from online risks that are not mutually exclusive but that reinforce and operate on each other. Moreover, this paper shows that facilitating digital resilience needs to be pursued as a collective endeavour that needs to be understood as a situated process.

With Computing being introduced into curricula in many countries, the need to support children to build and show digital resilience is increasingly important. However, evidence regarding how this can be optimally undertaken is lacking (Finkelhor et al., 2021; Sentance & Csizmadia, 2017). By understanding digital resilience within a socio-ecological framework and conceptualising it as a malleable process rather than an outcome (Southwick et al., 2014; Ungar, 2021), attention can become refocused on how children can be better supported individually, within homes, communities, and societies.

This is helpful when re-considering the effectiveness/ineffectiveness of universal Internet Safety Education and evidence indicating children with additional needs require more yet receive less help than their peers within educational settings (Hammond et al, 2022; Livingstone et al., 2017; Lundy et al., 2019). One-size-fits-all approaches devoid on contexts are likely to be suboptimal. Our socio-ecological framework represents the potential to maps strengths and areas of need across levels and processes to enable educators to across different areas to provide more targeted support.

Children are the internet’s most vulnerable users and simultaneously its pioneers (Harrison and Polizzi, 2021; Hammond et al, 2022). This is important for educators and schools, which are becoming increasingly positioned at the forefront of mitigating online risks and developing digitally literate citizens (OECD, 2021). In agreement with Livingstone (2013), this paper problematises default assumptions about online risks as inherently harmful. This reconceptualization offers an important starting point for educators and policy makers to reconsider their roles facilitating supported digital resilience opportunities across informal and formal education. Education seeks to provide learning environments, that is, spaces where digital skills and knowledge can be learnt, practiced, mistakes made and with tailored support and guidance improvements sought continuously over time (Stringer et al., 2022). Total risk avoidance within school settings is therefore undesirable. Hence, the ability to generate evidence on what works best, how, for whom, over what period, and at what cost represents an important next step (Pawson, 2013).

### Limitations

The paper has limitations. It does not offer specific guidance for educators or consider how digital resilience operates over time. Future work is needed to add this longitudinal understanding. Nor does the paper, despite highlighting the need to create child-centred opportunities to implement and optimise ‘one-size-fits-all’ digital resilience support, seek to comment on outcomes or prioritise one domain or level over another. Again, future work is needed to establish a valid, reliable and useable way to measure pre-teens’ digital resilience () from which this knowledge may be ascertained. We would suggest that any measure of digital resilience is orientated as a process measure rather than outcome measure to reflect the dynamic nature of the target phenomena.

Importantly, these limitations do not impact on the papers’ contribution, which is to reframe digital resilience as a socio-ecological concept. Whilst thick descriptions and insights offer transferability (as opposed to generalisations *per se*), this is only on a case-by-case basis, leaving home, key community, and societal actors to judge transferability to their own contexts (Lincoln & Guba, 1986). Clearly the qualitative methodology, sample size and setting impact generalisability. However, by blending rigor with richness (Lincoln & Guba, 1986), induction and abduction (Tavory & Timmermans, 2014), we offer a more nuanced conceptual understanding of digital resilience.

### Future directions

Our findings have the potential to help parents/carers and educationalists to promote digital resilience through formal and informal educational approaches that interact and show the importance of supporting pre-teens’ digital resilience. For policy makers, this study illustrates aspects that might otherwise be taken for granted and that can shape new ways of implementing ways to promote digital resilience. For researchers, we offer empirical evidence and conceptual understandings to inform future research.

The paper’s theoretical contributions are also important. In re-orientating digital resilience as situated across differing levels and processes, the paper enables theory to explain more closely the role of context. This problematizes knowledge of online risks as universally experienced as opposed to situated, contextual, and continuous phenomena (Livingstone et al., 2021; Stoilova et al., 2021). This is important as the role of the individual, family, community, and societal as social actors is brought into sharper focus. This will allow future research to examine the potential moderating role of digital resilience, in relation to risks experiences, digital literacy and their potential mental health consequences (Stoilova et al., 2021). This is important as, despite research indicating that risk exposure is a key precursor to digital resilience development (Stoilova et al., 2021; Sun et al., 2022; Vissenberg & d’Haenens, 2020; Vissenberg et al., 2022), no robust psychometric measurement of digital resilience exists, limiting the explanatory power and robustness of theory development (Vissenberg et al., 2022).

An important first step towards this goal is to build on the definition of digital resilience offered by UKCIS (2020). Based on our analysis we suggest that digital resilience may be better defined as: *a dynamic process whereby individuals and/or groups learn how to recognise, manage, and recover from online risks within and across individual, home, community, and societal levels.*

## Conclusions

In answering the question of how pre-teens’ digital resilience operates in ways that involve key actors, this paper illustrates how the development of digital resilience is a socio-ecological power-ridden process, mutually constituted by top-down and bottom-up forces, not, as previously emphasised, a solely psychosocial concept. This paper therefore provides a conceptual understanding of this process, which we hope can further understandings that improve how children are supported to develop digital resilience throughout their ecosystems.

## Data Availability

The data that support the findings of this study are available from the corresponding author, SH, upon reasonable request. The data are not publicly available yet in order to allow for further production of research outputs but will be available for reuse and archiving via the ESRC Data Store.

## Appendix 1

**Table:** COREQ (COnsolidated criteria for REporting Qualitative research) Checklist

Topic	Item No.		Guide Questions/Description		Reported on Page No.
**Domain 1: Research team and reflexivity**					
*Personal characteristics*					
Interviewer/facilitator	1		Which author/s conducted the interview or focus group?		5
Credentials	2		What were the researcher’s credentials? E.g. PhD, MD		8
Occupation	3		What was their occupation at the time of the study?		8
Gender	4		Was the researcher male or female?		8
Experience and training	5		What experience or training did the researcher have?		8
*Relationship with participants*					
Relationship established	6		Was a relationship established prior to study commencement?		7–8
Participant knowledge of the interviewer	7		What did the participants know about the researcher? e.g. personal goals, reasons for doing the research		7
Interviewer characteristics	8		What characteristics were reported about the inter viewer/facilitator? e.g. Bias, assumptions, reasons and interests in the research topic		9
**Domain 2: Study design**					
*Theoretical framework*					
Methodological orientation and Theory	9		What methodological orientation was stated to underpin the study? e.g. grounded theory, discourse analysis, ethnography, phenomenology, content analysis		5
*Participant selection*					
Sampling	10		How were participants selected? e.g. purposive, convenience, consecutive, snowball		7
Method of approach	11		How were participants approached? e.g. face-to-face, telephone, mail, email		7
Sample size	12		How many participants were in the study?		7
Non-participation	13		How many people refused to participate or dropped out? Reasons?		7
*Setting*					
Setting of data collection	14		Where was the data collected? e.g. home, clinic, workplace		6–7
Presence of non-participants	15		Was anyone else present besides the participants and researchers?		5
Description of sample	16		What are the important characteristics of the sample? e.g. demographic data, date		6–7
*Data collection*					
Interview guide	17		Were questions, prompts, guides provided by the authors? Was it pilot tested?		5
Repeat interviews	18		Were repeat inter views carried out? If yes, how many?		No
Audio/visual recording	19		Did the research use audio or visual recording to collect the data?		8
Field notes	20		Were field notes made during and/or after the interview or focus group?		No
Duration	21		What was the duration of the inter views or focus group?		6 & 8
Data saturation	22		Was data saturation discussed?		No
Transcripts returned	23		Were transcripts returned to participants for comment and/or correction?		8
**Domain 3: analysis and findings**					
*Data analysis*					
Number of data coders	24		How many data coders coded the data?		8–9
Description of the coding tree	25		Did authors provide a description of the coding tree?		No
Derivation of themes	26		Were themes identified in advance or derived from the data?		8–9
Software	27		What software, if applicable, was used to manage the data?		8
Participant checking	28		Did participants provide feedback on the findings?		No
*Reporting*					
Quotations presented	29		Were participant quotations presented to illustrate the themes/findings? Was each quotation identified? e.g. participant number		Yes
Data and findings consistent	30		Was there consistency between the data presented and the findings?		Yes
Clarity of major themes	31		Were major themes clearly presented in the findings?		Yes
Clarity of minor themes	32		Is there a description of diverse cases or discussion of minor themes?		Yes
